# Antimicrobial Activity of *Agastache* Honey and Characterization of Its Bioactive Compounds in Comparison With Important Commercial Honeys

**DOI:** 10.3389/fmicb.2019.00263

**Published:** 2019-02-25

**Authors:** Sushil Anand, Margaret Deighton, George Livanos, Paul D. Morrison, Edwin C. K. Pang, Nitin Mantri

**Affiliations:** ^1^The Pangenomics Group, School of Science, RMIT University, Melbourne, VIC, Australia; ^2^Kenkay Pharmaceuticals Pty Ltd., Smeaton Grange, NSW, Australia

**Keywords:** *Agastache* honey, Manuka, Jelly bush, *Leptospermum*, antimicrobial, LC-MS, methyl syringate, phenyllactic acid

## Abstract

There is an urgent need for new effective antimicrobial agents since acquired resistance of bacteria to currently available agents is increasing. The antimicrobial activity of Mono-floral *Agastache* honey produced from Australian grown *Agastache rugosa* was compared with the activity of commercially available honeys derived from *Leptospermum* species and with Jarrah honey for activity against clinical and non-clinical strains of *Staphylococcus aureus* (methicillin-susceptible and methicillin-resistant strains), *Pseudomonas aeruginosa*, and *Escherichia coli*. The minimum inhibitory concentration (MIC) for *Agastache* honey was in the range of 6–25% (w/v) for all species examined. The MICs for *Leptospermum* honeys were generally similar to those of *Agastache* honey, but MICs were higher for Super manuka and Jarrah honeys and lower for Tea tree honey. Staphylococci were more susceptible to all honeys than *Pseudomonas aeruginosa* and *Escherichia coli*. Pretreatment of honey with catalase increased the bacterial growth at MIC of Tea tree honey (35%), Super Manuka (15%), Jarrah honeys (12%), and *Agastache* honey (10%), indicating variable contributions of hydrogen peroxide to antimicrobial activity. Manuka and Jelly bush honeys retained their antimicrobial activity in the presence of catalase, indicating the presence of other antimicrobial compounds in the honey. An LC-MS/MS method was developed and used to identify possible antimicrobial phenolic compounds in *Agastache* honey and flowers, and five commercial honeys. The chemical markers characteristic of *Agastache* honey and honeys of *Leptospermum* origin were phenyllactic acid and methyl syringate. Overall, the bioactive compounds with antimicrobial and antioxidant activity in *Agastache* honey suggested a possible use for topical application and in wound care.

## Introduction

The well-known antimicrobial activity of honey and its recent use in clinical settings has reinvigorated further investigation of bioactive honeys i.e., honeys marketed as having therapeutic potential. Some honeys show broad-spectrum activity against antibiotic-resistant bacteria (Wang et al., 2012), while others are very effective against biofilm forming clinical isolates of methicillin resistant *Staphylococcus aureus* (MRSA) and *Pseudomonas aeruginosa* (Alandejani et al., 2009). Honey was shown to be effective in alleviating inflammation associated with wound infections and enhancing healing (Efem, 1988; Yaghoobi et al., 2013). Honey dressing was effective in decreasing morbidity associated with first and second degree burns and assisting in reducing the time required for rehabilitation (Baghel et al., 2009; Wijesinghe et al., 2009). Lund N.B.et al studied the effect of Manuka honey-coated bandages compared with silver-coated bandages on treatment of malignant wounds and reported that honey coated bandages were effective similar to the silver-coated bandages in reducing the size of malignant wounds (Lund-Nielsen et al., 2011).

Honey constitutes 81% sugar, 17% water, and 1–2% of other enzymes and compounds (White, 1957; Jeffrey and Echazarreta, 1996). These 2% of remaining compounds are important contributors to the bactericidal activity of the honey and their composition determines the variability of honey (Molan, 1992; Kwakman and Zaat, 2012). Part of the antibacterial activity of honey is due to hydrogen peroxide, but the level of hydrogen peroxide varies considerably depending on the floral source and the environmental conditions under which the plant has been grown. Some honeys retain antibacterial activity even when hydrogen peroxide is neutralized with catalase. This indicates presence of other antimicrobial compounds. These differences are reflected in the minimal inhibitory concentration (MIC) required to inhibit various bacteria, which may range from concentrations of <3% to 50% and higher (Carter and Thornburg, 2004). Several honeys, for example Munuka honey, have been identified as having non-peroxide based antibacterial activity (Irish et al., 2011). Commercially important honeys such as Australian Jelly bush honey and Jarrah honey have also been marketed as bioactive honeys. Factors, in addition to hydrogen peroxide, that contribute to the antibacterial activity of these honeys are osmotic pressure, pH, bee-defensin-1 peptide, and phenolic compounds (Kwakman and Zaat, 2012).

Phenolic compounds or poly-phenols, which include phenolic acids and flavonoids, are secondary metabolites which are widely distributed in plants. These compounds are derivatives of the pentose phosphate, shikimate, and phenylpropanoid pathways. Over 5,000 such compounds have been described (Dimitrova et al., 2007). Generally, phenolic compounds have been classified into three groups: flavonoids, cinnamic acids, and benzoic acids (Pyrzynska and Biesaga, 2009). Some researchers categorize phenolic acids into phenolic esters and flavonoids. The phenolic compounds in the nectar honey include free phenols (volatile compounds), phenolic acids, polyphenols (usually in the form of flavonoids), anthocyanins, procyanidins, and pigments (Carson, 2000).

A wide variety of health benefits have been attributed to the phenolic compounds. These include antimicrobial, antioxidant, anti-inflammatory, anti-allergenic, anti-thrombotic, cardio-protective, and vasodilatory effects (Vichapong et al., 2010). The specific beneficial effects of any honey depend on its phenolic composition, which in turn is related to the nectar composition of the particular plant species. Indeed, the phenolic composition of any honey is regarded as an important determinant of its floral and geographical origin. For example, citrus honey is identified by the flavanone hesperetin, while rosemary and sunflower honeys typically contain flavonols, kaempferol, and quercetin (Ferreres et al., 1993; Gil et al., 1995). Chemical markers used to identify other honeys are given in Table 1. These markers are specific to the plant species and their concentration is dependent on the phenolic composition of the nectar.

**Table 1:** Chemical markers assigned to honeys.

**Honey type**	**Chemical markers**	**References**
Eucalyptus honey	Myricetin, tricetin, and luteolin	Martos et al., 2000a
Acacia honey	Kaempferol–rhamnosides and rhamnosyl–glucosides	Truchado et al., 2008
Heather honey	Myricetin, Myricetin-3-methyl ether, tricetin	Ferreres et al., 1994
Chestnut honey	p-coumaric and ferulic acids	Alissandrakis et al., 2011
Polish yellow sweet clover honey.	Caumarin	Oomah and Mazza, 1996
Polish heather and buckwheat honey	Abscisic acid	Jasicka-Misiak et al., 2012
Sage honeys	p-coumaric, p-hydroxybenzoic, and ferulic acid	Kenjerić et al., 2008

In the last decade, tandem mass spectrometry has been used to identify chemical markers specific to honeys from different parts of the world. Ion trap and triple quadrupole mass spectrometers and other hyphenated methods have been used to characterize the phenolic profile of plant extracts and honey samples. This study reports for the first time the antimicrobial activity of *Agastache* honey and its bioactive compounds. The *Agastache* honey was produced from a single floral source in a closed glass house and its physicochemical properties and antioxidant capacities were reported recently (Anand et al., 2018). The present study is an extension of the previous study, which demonstrated significant antioxidant capacity of *Agastache* honey. The main aims of the present study were to (i) compare the antimicrobial activity of *Agastache* honey with that of commercially available bioactive honeys, namely *Leptospermum* honeys (Australian Tea tree honey, Jelly bush honey, Super manuka honey), Australian Jarrah honey and New-Zealand Manuka honey, (ii) characterize and compare the phenolic profile of *Agastache* honey and flowers (containing nectar) with those of the commercially available bioactive honeys, (iii) identify compounds in the honeys with possible antimicrobial activity.

## Materials and Methods

### Honey Samples

*Agastache* honey was produced as described previously (Anand et al., 2018). Manuka honey (hnz, UMF 22+, *Leptospermum scoparium*), Tea-tree honey (Miellerie, *Leptospermum lanigerum and Leptospermum scoparium*), Jelly bush honey (Australia's Manuka, 20+ Active, *Leptospermum polygalifolium*), super manuka honey (Berringa, MGO-400*, Leptospermum polygalifolium*) and Jarrah honey (Elixir, TA 45+ *Eucalyptus marginata*) were purchased from a commercial outlet.

### Minimum Inhibitory Concentration (MIC) and Minimum Bactericidal Concentration (MBC) Determination

The bacterial strains used in this study were methicillin-susceptible *Staphylococcus aureus* (MSSA) ATCC 25923, methicillin-resistant *S. aureus* (MRSA) ATCC BAA-1698, MRSA clinical isolate I, MRSA clinical isolate II, *Escherichia coli* ATCC-11560, *E. coli* clinical isolate I*, Pseudomonas aeruginosa* ATCC-21853, and *P. aeruginosa* clinical isolate I. The clinical isolates, from infected wounds and skin, were obtained from Dorevitch Pathology, Melbourne.

Antimicrobial activity was evaluated by determining the Minimum Inhibitory Concentration (MIC) and Minimum Bactericidal Concentration (MBC) of the honeys. The assays were performed in sterile, 96-well, round bottom, polystyrene microtitre plates (Corning Coster Ltd., NY) according to the CLSI guidelines modified by Wiegand et al. (2008). Briefly, bacteria were streaked onto nutrient agar plates and incubated for 24 h. Colony suspensions were prepared by touching 3–5 colonies with a sterile loop and transferring the inoculum into sterile 3-5 ml Mueller Hinton Broth (MHB). Turbidity was adjusted to 0.5 McFarland with sterile MHB, then suspensions were further diluted 1:100. Honey suspensions (50%) were prepared in sterile MHB, filtered through 0.45 μM filters and ten two-fold dilutions were made. Fifty Microliter volumes of the diluted bacterial suspensions were inoculated into wells containing the honey dilutions resulting in the final inocula of ~5 × 10^5^ cfu/ml.

Sterility controls containing media only and growth controls containing bacteria only were included in the assay. Inoculum size was validated by removing 10 μl volumes from the growth control well and performing viable counts. To control for the color of different honey samples, OD_600_ measurements were taken at 0 h and after 24 h incubation at 37°C in the dark. Growth of bacteria in the presence of honey was assessed by the following formula: OD_honeytreatedwells_/ OD_negativecontrolwell_ × 100, where the control well was assigned 100% growth. To assess bactericidal activity, 10 μl samples from the first 2 wells containing no visible growth were plated onto the nutrient agar plates and incubated for 24 h. The bacteriostatic end-point was defined as the highest dilution showing growth inhibition. The bactericidal end-point was defined as the highest dilution showing no growth on the inoculated plates.

The effect of *Agastache* honey on bacterial cell viability was further investigated by Laser Confocal microscopy. Two fluorescent dyes were used in combination: SYTO9 and PI (Invitrogen AG, Basel, Switzerland). Stock solutions of the dyes were prepared as follows: PI and SYTO9 were used from the LIVE/DEAD BacLight kit (Invitrogen) as instructed by the manufacturer. All stock solutions were stored at −20°C. Briefly, equal volumes of Component A and Component B were mixed thoroughly in a microfuge tube. About 3 μL of the dye mixture for each mL of the bacterial suspension (after 24 h of treatment) was added. The sample was mixed thoroughly and incubated at room temperature in the dark for 15 min. Subsequently, 5 μL of the stained bacterial suspension was placed between the slide and an 18 mm square cover slip and observed under 60X magnification in a Nikon Confocal microscope (Nikon Eclipse TS100, Japan) at wavelength 488–620 nm (PI:535/617; SYTO9:488/498).

### Antimicrobial Activity After Inactivation of Catalase

The hydrogen peroxide activity of honey was eliminated by treating dilutions of honey with catalase (Sigma, C-40) at a final concentration of 0.2% w/v (Allen et al., 1991), prior to MIC determinations, using *E. coli* clinical isolate I as the test strain. Sterility control wells received broth and catalase only, while the growth control received bacteria, broth and catalase. Plates were incubated as described in the previous section and MIC and MBC values were determined.

### Statistical Analysis

Minitab 18 (MiniTab Inc., PA, USA) was used to compare the bacterial growth after treatment with different concentrations of honey. The amount of the bacterial growth was transformed into Log_10_ values. An alpha level of 0.05 was assumed for the determination of statistical significance. Analysis of variance (ANOVA) was followed by *post hoc* Tukey test and Kristal Wallis tests. Data were calculated from three different experiments each conducted in triplicate.

### HPLC-ESI-MS/MS Analysis

#### Reagents and Materials

Gallic acid, protocatechuic acid, catechin, 4-hydroxybenzoic acid, chlorogenic acid, caffeic acid, phenyllactic acid, 2,4- dihydroxybenzoic acid, vanillic acid, syringic acid, p-caumaric acid, ferulic acid, rutin, sinapic acid, cinnamic acid, rosmarinic acid, methyl syringate, quercetin, hesperetin and kaempferol were obtained from Sigma Aldrich (Australia). HPLC grade methanol was supplied by Merck (Australia). Stock solutions of the compounds were prepared by dissolving 5 mg in methanol. Phenomenex (Australia) Strata-X SPE cartridges (500 mg/6 ml, surface area 800 m^2^ g^−1^, particle size 33 μm, average pore size 85 Å) were used to pre-concentrate the target analytes. SPE cartridges were conditioned according to the manufacturer's instructions before loading the honey samples.

#### Analytical Conditions

The honey extracts were subjected to liquid chromatography/tandem mass spectrometry (LC-ESI-MS/MS). The system was an Agilent 1200 liquid chromatograph and a 6410 triple quadrupole mass spectrometer with an electrospray ionization source. Data were analyzed using the MassHunter software package (Agilent Corporation, MA, and USA). Separation was performed on a Zorbax C18 column (2.1 mm × 50 mm, 0.18 μm particle size) by gradient elution using the following gradient: 0–1 min 5% B isocratic; 1–14 min linear gradient to 100% B; 14–15 min to 5% B and the column was reconditioned at 5% B for 5 min. Eluent A was water with 0.1% formic acid and B was methanol with 0.1% formic acid. The acid used in the analysis improves the chromatographic peak shape and provides a source of proton in reverse phase LC-MS. The column temperature was 30° C. Mass spectrometry was performed on triple quad spectrometer in ESI positive ion mode. High purity nitrogen served as both the nebulising agent and the drying gas, with a drying gas flow 9 l/min and a nebuliser pressure 25 psi at 350°C. The capillary voltage was set at 4,000 V and the source at 300°C.

#### Extraction of Phenolic Compounds

The harvested *Agastache* honey and other honey extracts were prepared by dissolving 5 g of honey into 50 ml of acidified water (pH-2) as described by Michalkiewicz et al. (2008). The 10% aqueous honey solutions were stirred for 15 min then filtered through Whattman paper 1. The extracts were loaded onto Strata-X cartridges and passed through the cartridge at 2 ml/min with the help of vacuum pump. Generally, the phenolic compounds become attached to the sorbent (500 mg) after loading honey extracts. The remnants of non-phenolic compounds or unbound phenolic compounds were washed with 10 ml of acidified water (pH-2). The attached phenolic compounds were eluted with 10 ml of methanol and dried down to 1 ml under the flow of nitrogen stream. Samples were lyophilised since the cartridge retained some water during the process and as a result water eluted with samples. The lyophilised samples were resuspended in 10% methanol before injecting into LC-MS/MS for the analysis. All samples were analyzed in triplicate.

The phenolic compounds were extracted from *Agastache* flowers with 50% methanol as described by Wijekoon et al. (2011) with certain modifications. Inflorescences of *Agastache rugosa* were obtained from RMIT Herbal garden. Fresh inflorescences were collected and freeze dried for 48 h. On completion of freeze drying, the samples were ground to a fine powder and stored at 4°C. Five grams of accurately weighed freeze dried sample powder was mixed with 200 ml of (50%) methanol and extracted in a reagent bottle (covered with aluminum foil) overnight at room temperature. Extracts were filtered using Whatman No. 1 filter paper. The remaining residue on the filter paper was transferred back into the same reagent bottle and re-extracted until the residue became colorless. The pooled filtrates were concentrated under reduced pressure in a rotavapor (Buchi rotavapor 461) to obtain solvent free crude extracts. Following removal of methanol, filtrates were lyophilized and samples were prepared as described above.

#### Method Validation

All reference stock solutions of standard phenolic compounds were prepared in methanol at 5 mg/ml concentration and diluted to 10% methanol solution. The five level calibrators were prepared in the range from 0 to 25 μg/ml to construct calibration curves. To plot calibration curve of responses vs. concentration of analytes, the Agilent MassHunter Quantitative Analysis Software was used. For a measure of precision and accuracy, relative standard deviations (RSD) were calculated. To evaluate repeatability and reproducibility of the method, intra-day and inter-day precisions were determined. For intra-day determinations, samples were analyzed in pentaplicate, while samples were analyzed over seven consecutive days for inter-day precision. For the recovery experiment, the standards (25 μg/ml) were spiked onto artificially synthesized honey and loaded onto SPE-X cartridges as described above. The artificially synthesized honey, comprising 32 g of fructose, 31 g of glucose, 12 g of maltose, and 0.1 g of sucrose in 100 ml of distilled water, was sterilized at 121°C for 15 min (Sherlock et al., 2010).

## Results

Minimum Inhibitory Concentration (MIC) and Minimum Bactericidal Concentration (MBC) determination.

**Figure 1:**
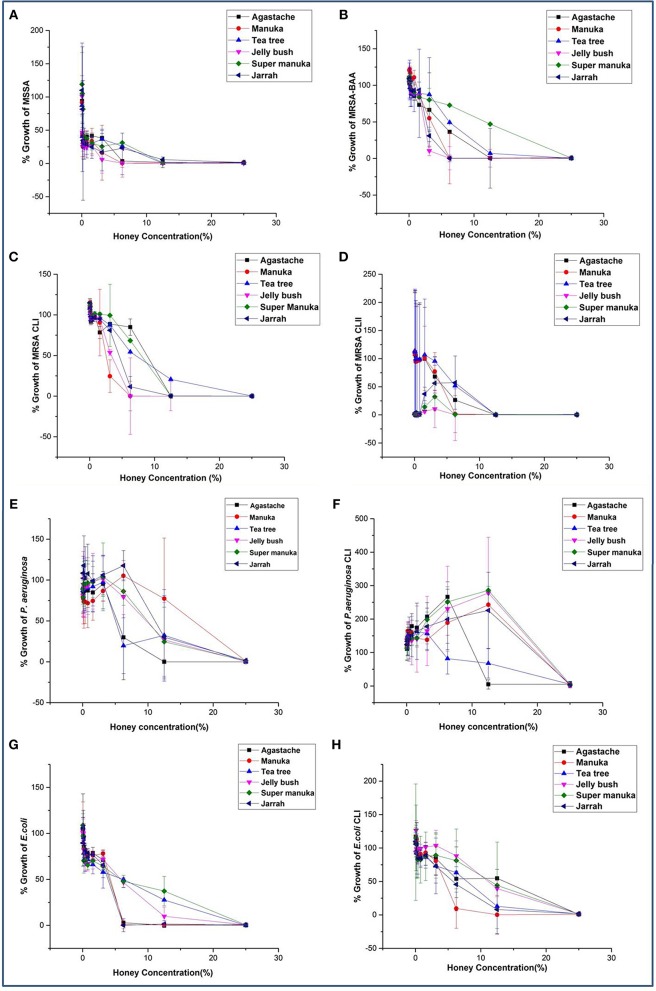
**(A–H)** Antimicrobial activity of honey on bacterial species known to cause superficial skin infections.

All honeys showed some antibacterial activity. The MICs and MBCs of six honeys against eight bacterial reference strains including clinical isolates varied, depending on the bacterial species, strain and the type of honey (Table 2, Figures 1A–H). In general, all honeys were more effective against staphylococci than against Gram-negative bacteria. MICs and MBCs against staphylococci differed by no more than one dilution, indicating bactericidal activity. In contrast, higher concentrations of most honeys were required to achieve bactericidal activity against Gram negative bacteria. *P. aeruginosa* was slightly more resistant to most honeys than *E. coli*. Tea tree honey was more efficacious than other *Leptospermum* origin honeys (Manuka, Jelly bush and Super manuka).

**Table 2:** Minimum inhibitory concentration (MIC) and minimum bactericidal concentration (MBC) of honey (%) to inhibit 100% of the microbial growth *in vitro* expressed in % w/v solution (*n* = 3).

**Bacterial strains**	***Agastache***		**Manuka**		**Super manuka**		**Tea tree**		**Jarrah**		**Jelly bush**	
	**MIC**	**MBC**	**MIC**	**MBC**	**MIC**	**MBC**	**MIC**	**MBC**	**MIC**	**MBC**	**MIC**	**MBC**
MSSA	6.25	12.5	6.25	12.5	12.5	12.5	6.25	6.25	12.5	12.5	12.5	12.5
MRSA-BAA	12.5	12.5	6.25	6.25	12.5	25	3.12	3.12	25	12.5	3.12	6.25
MRSA CI-I	12.5	12.5	6.25	12.5	25	12.5	6.25	6.25	12.5	12.5	12.5	12.5
MRSA CI-II	6.25	6.25	6.25	12.5	6.25	12.5	3.12	6.25	12.5	25	3.12	12.5
*Ps.aeruginosa*	6.25	25	25	25	12.5	25	6.25	25	12.5	25	12.5	25
*Ps aeruginosa* CI-I	12.5	25	25	25	25	25	12.5	25	25	25	25	25
*E.coli*	6.25	12.5	6.25	12.5	25	25	12.5	25	25	25	6.25	12.5
*E.coli* CI-I	25	25	6.25	25	25	25	12.5	25	25	25	12.5	25

Statistical analysis showed that honey concentration, honey type and bacterial strain affected growth of all bacteria and all interactions between these three parameters were significant. The main influence on activity was honey concentration, which explained 65% of the variation, bacterial strain (12% of the variation), while honey type and all interactions explained less than 3% each (Table 3). Bacterial growth was reduced only by the critical honey concentration of >3.12%, and concentrations less than 3.12% had no effect on the bacterial growth. The non parametric KW test also showed that honey concentration had a significant effect on bacterial growth (*p* = 0.001) but honey type was not significant (*p* = 0.09).

**Table 3:** Analysis of variance (ANOVA) main effect of independent variables: tests of independent variables.

**Source**	**DF**	**Adj SS**	**Adj MS**	***F*-Value**	***P*-Value**
Honey concentration	10	565.748	56.5748	378.29	0.001
Honey type	6	8.203	1.6406	10.97	0.001
Bacterial strain	8	54.014	7.7163	51.59	0.001
Honey concentration*Honey type	60	26.230	0.5246	7.08	0.001
Honey concentration*Bacterial strain	80	67.405	0.9629	13.00	0.001
Honey type*Bacterial strain	48	9.582	0.2738	3.70	0.001
Honey concentration*Honey type*Bacterial strain	480	60.781	0.1737	2.34	0.001
Error	1056	78.223	0.0741		
Total	1583	866.844			
S	R-sq	R-sq(adj)	R-sq(pred)		
0.272167	90.98%	86.47%	79.70%		

**Figure 2:**
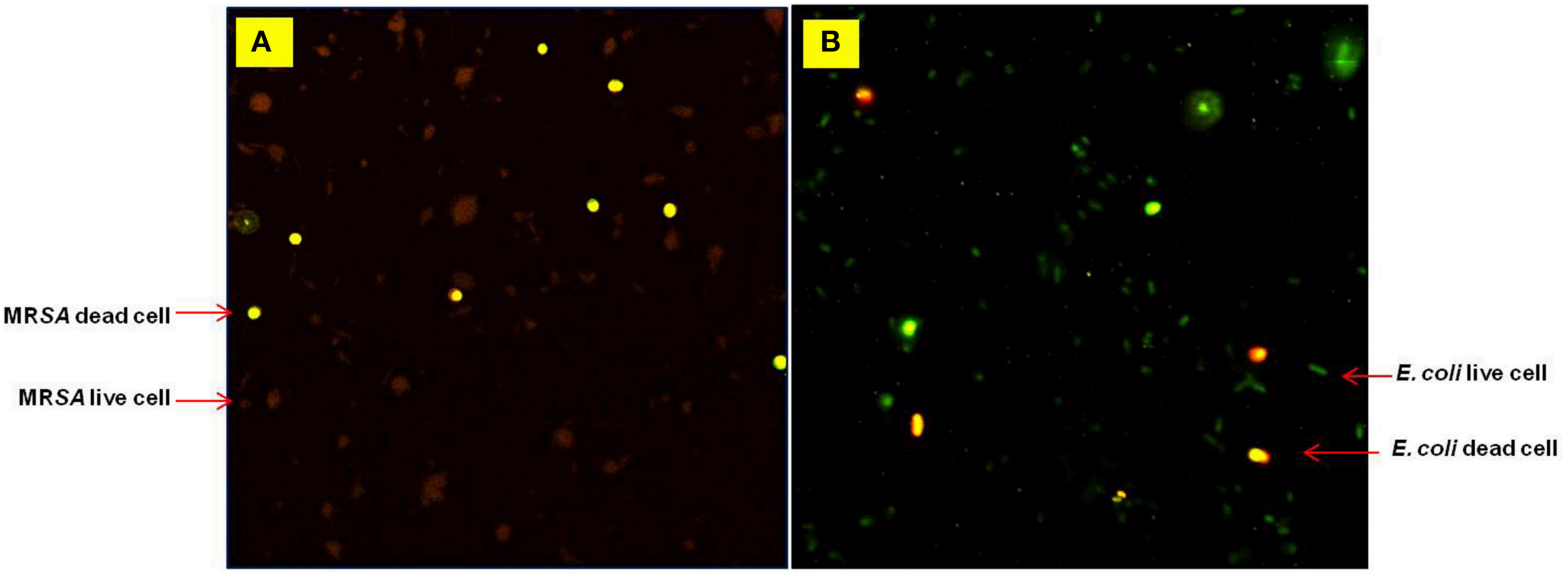
Confocal microscopy images obtained during a Live/Dead cells assay for **(A)** MRSA-BAA at 6.25% and **(B)** *E. coli* at 12.5% (w/v) *Agastache* honey. Live cells (stained green) Dead cells (stained red).

Effect of *agastache* honey on the viability of bacterial strains such as MR*SA* and *E. coli* was further analyzed by confocal microscopy. The viability of bacterial cells treated with different concentrations of *Agastache* honey was analyzed at concentration one dilution lower than MIC: 6.25% for MR*SA*-BAA and 12.5% for *E. coli* clinical isolate I (Figure 2). Spatial difference between the treated and untreated samples was also observed. Analysis of confocal images at higher concentrations of honey showed extensive cell lysis and membrane disruption; at lower magnification, bacterial lysis throughout the entire sample was visible and there was a large amount of extracellular material visible. We determined the contribution of hydrogen-peroxide to the antimicrobial activity of the different honeys by inactivating hydrogen-peroxide with catalase prior to MIC and MBC determinations. Following inactivation, bacterial growth at higher concentrations of honey (25%, 12.5%, 25%, 25%) corresponded to MIC values for *Agastache*, Tea tree, Super manuka and Jarrah honeys were monitored. This is in agreement with previous observations that Manuka and Jelly bush honey showed no variation in the bacterial growth at MICs in the presence and absence of hydrogen peroxide (Mandal and Mandal, 2011). However, a change was observed in the bacterial growth at honey concentrations of 6.25% (data not shown). The bacterial growth at MIC of tea tree honey increased in the presence of catalase to a greater extent (35%) than the MICs of all other honeys, indicating that the antibacterial activity of this honey is largely due to hydrogen peroxide. Notably, the bacterial inhibition at MIC of *Agastache* honey dropped by 10% when hydrogen peroxide activity was eliminated by treatment with catalase, indicating a small contribution of hydrogen peroxide to the antimicrobial activity of *Agastache* honey.

### HPLC-ESI-MS/MS Analysis: Method Development and Evaluation

The LC-MS/MS method was developed successfully to analyse phenolic compounds commonly found in honey using a standard system (Agilent-6410). Initially, the performance of the instrument i.e., the highest signal to noise ratios was determined by scanning samples in positive ion and negative ion mode. In the initial scan (LC-MS scan) of analytes of interest in the positive ion mode, resolution of the peaks was better than in the negative ion mode. The MS spectra obtained in this mode were dominated by the [M+H]^+^ precursor ions. The analytes were further fragmented to obtain characteristic fragmentation patterns for the individual compounds. The collision energy, an instrument parameter which is used to increase the ion intensity and is responsible for generating fragmentation patterns for each compound, was determined. Initially, each phenolic compound was broken into fragments with collision energy of 20 eV. However, CE for some compounds was optimized with a pair of identified precursor/product ions (Table 4). On the basis of precursor ions and product ions, these compounds were identified and quantified using the Multiple Reaction Monitoring (MRM) analysis mode. Many authors have analyzed the fragmentation patterns of phenolic compounds in the negative ion mode (Pyrzynska and Biesaga, 2009; Sergiel et al., 2014), but the patterns in positive ion mode have not been reported previously.

**Table 4:** LC-MS/MS parameters for detection of phenolic compounds in MRM mode.

**Standard phenolic compounds**	**Molecular mass**	**Precursor ion [M+H]^**+**^**	**Product ions (% relative abundance)**	**CE (eV)**	**Retention time**
Gallic acid	170.1	171.0	109.2 (100), 107 (71.55)	15	1.37
Protocatechuic acid	154.1	155.0	65.3 (100), 92.9 (42.8)	20	2.92
4, hydroxybenzoic acid	138.1	139.1	77.1 (100), 95.1 (67.7)	20	5.04
Catechin	290.2	291.1	139.2 (100), 123.1 (50)	20	5.90
2,4, dihydroxybenzoic acid	154.1	155.1	137.1 (100), 81.3 (20.36)	20	5.97
Chlorogenic acid	354.3	355.1	163.2 (100), 145.1 (11.1)	20	6.24
Vanillic acid	168.1	169.1	65.1 (100), 93.2 (72.7)	20	6.27
Caffeic acid	180.1	181.1	117.1 (100), 135 (72.6)	20	6.42
Syringic acid	198.1	199.1	140.1 (100), 95.1 (15.4)	20	6.81
Phenyllactic acid	166.1	167.1	103.2 (100), 79.2 (19.8)	20	7.29
P-coumaric acid	164.1	165.1	119.1 (100), 91.1 (85.7)	20	7.36
Ferulic acid	194.1	195.1	145.1 (100), 117.1 (96.6)	20	7.69
Sinapic acid	224.2	225.2	119.1 (100), 91.1 (66)	20	7.77
Rutin	610.5	611.5	303.1 (100), 85.1 (9.3)	20	8.38
Methyl syringate	212.1	212.9	154.2 (100), 181.2 (79.2)	20	8.43
Rosmarinic acid	360.3	361.3	163.2 (100), 135.2 (60.2)	30	8.62
Cinnamic acid	148.1	149.1	103.1 (100), 131 (21.2)	20	9.20
Quercetin	302.2	303.1	153.2 (64.15), 229.1 (63.1)	30	9.58
Hesperetin	302.2	303.2	153.1 (100), 117.1 (28.7)	35	9.58
Kaempferol	286.2	287.2	153.1 (100), 121.1 (63.5)	35	10.5

The HPLC gradient program was developed to separate phenolic acids and flavonoids in a short span of time. The retention time and elution order of the compounds are given in the Table 4. Most of the phenolic compounds eluted separately except hesperetin and quercetin. However, these co-eluting compounds were characterized by individual fragmentation patterns, which enabled them to be identified and quantified in the MRM analysis mode.

Method validation involved tests for linearity, precision, accuracy and recovery. For linearity, the phenolic compounds were tested in the concentration range of 0–25 μg/ml, The response obtained for analytes in the MRM mode was linear with determination co-efficient (*R*^2^) > 0.90. For reproducibility of signals, samples were injected for 5 times consecutively and expressed as %RSDs. The calculated %RSD values were in the range of 0.31–4.3% except for rosmarinic acid (12.41%) while, intra-day % RSD values was in the range of 0.57–7.45% except for rosmarinic acid (17.01%).

**Figure 3:**
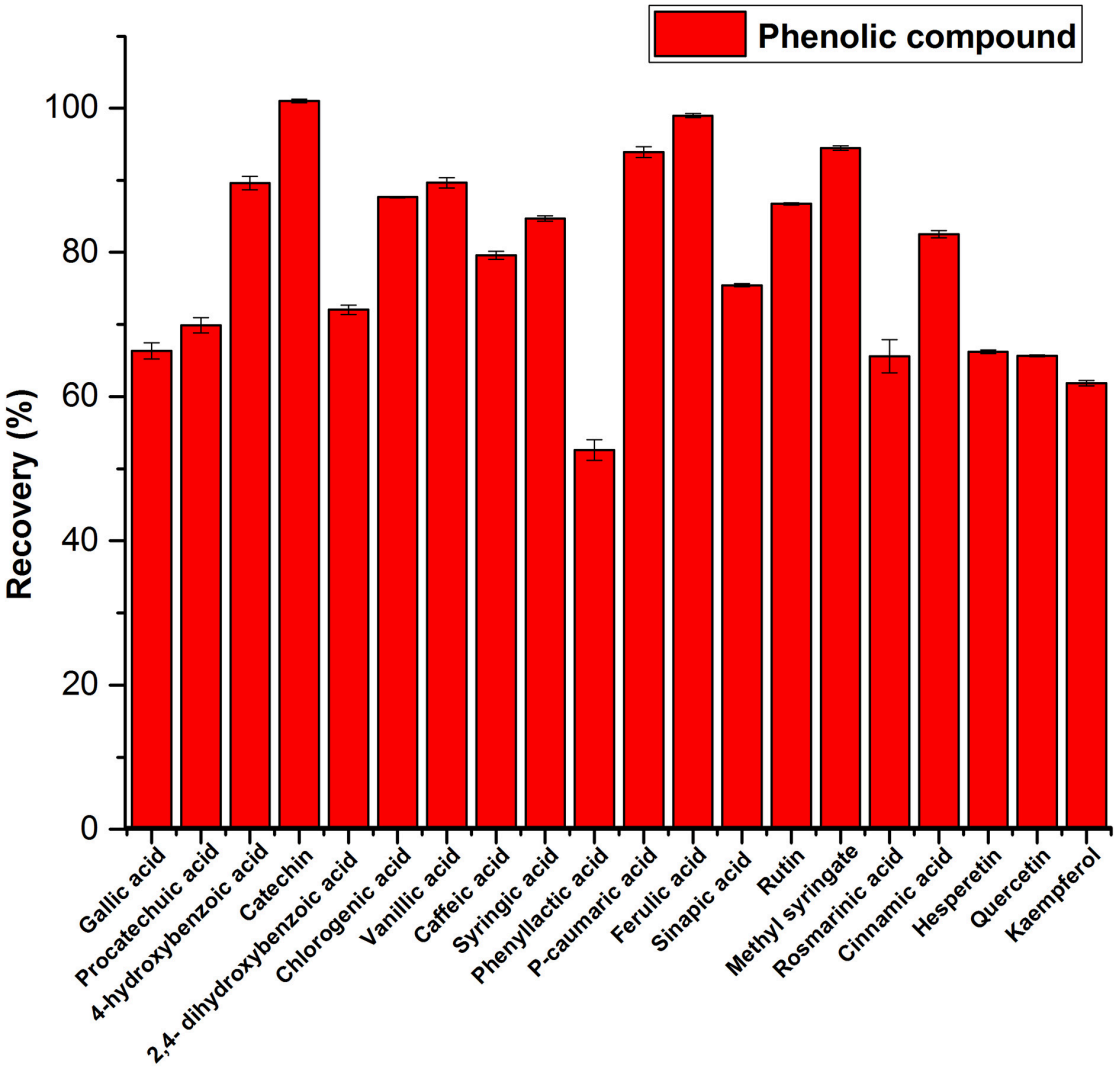
Recovery of phenolic compounds from spiked artificial honey.

Most of the compounds including flavonoids were recovered from spiked artificial honey (Figure 3). The recovery of all compounds was in the range of 52.56–100%. Phenyllactic acid was the least recovered compound and catechin was the most recovered at 100%. This indicates that the quantified amount of the compounds in the honey samples are likely to be underestimated.

### Identification of Compounds

Twenty of the phenolic compounds were screened and identified in honey samples using fragmentation patterns obtained by MS/MS analysis. Each compound exhibited a characteristic fragmentation pattern, which could be due to a loss of hydroxyl, carboxylate, carbonyl, alkoxy group, methyl group and fission of the oxy-aryl ring. Some compounds undergo protonation during the ionization process, and these protonated species do not undergo complete fragmentation. However, an increase in the collision energy tends to favor complete fragmentation of the protonated molecule. The relative abundance of these fragments was highly associated with the applied collision energy. The basic fragments produced by optimized collision energy is explained here.

The fragmentation ions generated for phenolic compounds are given below; the characteristic fragmentation ions generated for gallic acid were at *m/z* 153.1, 109.1, and 107.1 due to loss of hydroxyl group, carboxylate group and two hydrogen atoms, respectively. The fragments were of high abundance at *m/z* 107.1 and 109.1, which was due to their stability of those fragments.

The proto-catechuic acid showed its fragmentation components at *m/z* 137.1, 93.1, and 65.1 due to loss of hydroxyl, CO_2_ (or COO^−^), and carbonyl (or CO moiety specifically) group, respectively. Catechin showed an abundant fragment ions originated from Retro-Diels-Alder (RDA) fragmentation in the aryl ring, responsible for the appearance of the major fragment ions observed at *m/z* 139.1 and 123.2 (due to loss of O atom).

4-hydroxybenzoic acid exhibited a characteristic fragment ions at *m/z* 95.1 and 77.1 due to a loss of COO^−^ and hydroxyl group, respectively. Chlorogenic acid exhibited a characteristic major fragment ions at *m/z* 163.2 and 145.1 due to a loss of quinic acid (198.1) and hydroxyl group. The loss of quinic acid is the characteristic fragmentation pattern of the chlorogenic acid (Hossain et al., 2010). Due to decarboxylation of phenyllactic acid, the fragment ion at *m/z* 121.2 and a loss of hydroxyl and H atom, fragment ion at *m/z* 103.2 were observed. Fragment ions generated at *m/z* 79.2 remained unexplained.

The fragment ions produced for caffeic acid was at *m/z* 135.1 due to decarboxylation and fragment ions observed at m/z at 117.1 and 89.2 were due to a loss of hydroxyl and the ethylene moieties. 2,4-dihydroxybenzoic acid tends to lose hydroxyl and CO/CHO groups during fragmentation, which was evident from the formation of the fragment ions at *m/z* 137.1, 108.9, and 81.3. The removal of a hydroxyl group from the vanillic acid produced fragment ions at *m/z* 150.9 followed by the loss of carbonyl group which resulted in fragment ion formation at *m/z* 123.2. A fragment ion at *m/z* 93.2 was due to a loss of methoxy group with rearrangement or addition of a hydrogen atom to the compound followed by the removal of carbonyl group resulting in formation of fragments at *m/z* 65.1.

Syringic acid generated fragment ions at *m/z* 181.1, 155.2, 140.1, and 95.1 due to loss of hydroxyl, C_2_H_2_, methyl group, and carboxyl group, respectively. Removal of a hydroxyl group from *p*-caumaric acid produced fragment ion at *m/z* 147.1, followed by a loss of carbonyl groups which resulted in fragments at *m/z* 119.1 and 91.2.

Ferulic acid fragment ions observed at *m/z* 177.1, 145.1, 117.1, and 89.2 were produced as the result of loss of hydroxyl, methoxy, and carboxyl groups respectively. Rutin was cleaved at the interglycosidic bond and produced a fragment ion at *m/z* 303.1 and another fragment ion was produced at *m/z* 85.1. Sinapic acid was characterized by fragments ions at *m/z* 207.2, 192.1, 147.2, 119.2, and 91.2 due to loss of methyl, carboxyl groups and CO groups, respectively.

Rosmarinic acid exhibited fragmentation ions at *m/z* 163.2 due to loss of 2-hydroxy derivative of 2-hydrocaffeic acid as reported previously (Hossain et al., 2010). Fragmentation peaks were observed at m/z 135.2 and 89.2 due to a loss of carbonyl groups and 117.2 due to a loss of hydroxyl group. For cinnamic acid, fragmentation ions at *m/z* 131.1, 103.1, and 77.1 were due to a loss of a water molecule, carbonyl group and C_2_H_2_, respectively.

**Figure 4:**
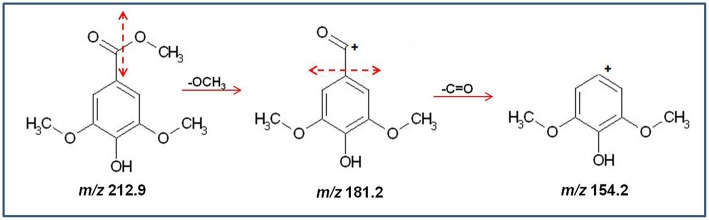
Schematic diagram of the production of major fragments from Methyl Syringate (*m/z* 212.9).

The fragmentation pattern of methyl syringate showed the loss of alkoxy group by alpha cleavage from the molecular ion resulting in formation of an acylium ion at *m/z* 181.2, followed by the loss of a carbonyl group generating fragments at *m/z* 154.2 (molecular ion with the addition of hydrogen ion) (Figure 4).

Quercetin showed a characteristic fragment ions at *m/z* 285.1, 257.2, 229.2, 201.2, and 153.01 due to loss of hydroxyl, carbonyl groups, and C_5_H_4_O, respectively. Kaempferol exhibited major fragment ions at 153.1 and 137.1 due to cleavage in the beta ring of the structure; however, other small fragment ions were also observed. Similarly, hesperetin showed a fragmentation ion at *m/z* 153.1 due to cleavage in the beta ring. A fragment ion at *m/z* 89.2 was obtained due to the loss of carbonyl group, but another fragment ion at *m/z* 117.1 was unexplained.

### Bioactive Compounds in *Agastache* Honey

**Figure 5:**
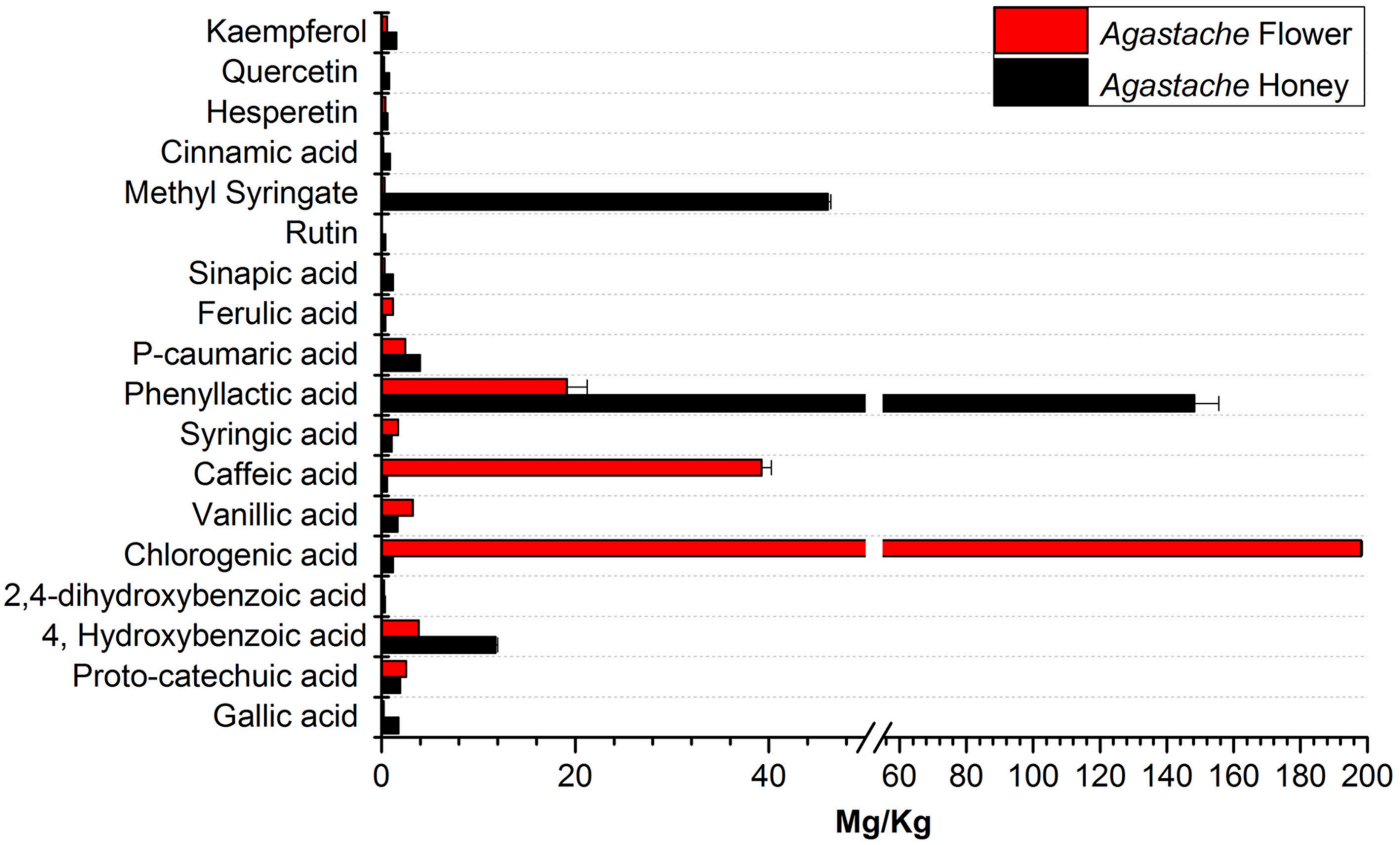
Comparative analysis of phenolic compounds in *Agastache* honey and *Agastache* flower.

The phenolic compounds profile of *Agastache* honey and flower extracts showed presence of a variety of compounds (Figure 5). Compounds common to honey and flower included proto-catechuic acid, 4-hydroxybenzoic acid, 2,4-dihydroxybenzoic acid, chlorogenic acid, vanillic acid, caffeic acid, syringic acid, phenyllactic acid, *p*-caumaric acid, ferulic acid, sinapic acid, methyl syringate, cinnamic acid, hesperetin and kaempferol. However, there were considerable differences in the amounts of most compounds in the two sites, for example, phenyllactic acid and methyl syringate were present at very high concentrations in honey, but smaller concentrations were present in flower. In contrast, caffeic acid and chlorogenic acid were present in large amounts in flower, but only low concentrations in honey.

### Bioactive Compounds in Honey of *Leptospermum* Origin

**Figure 6:**
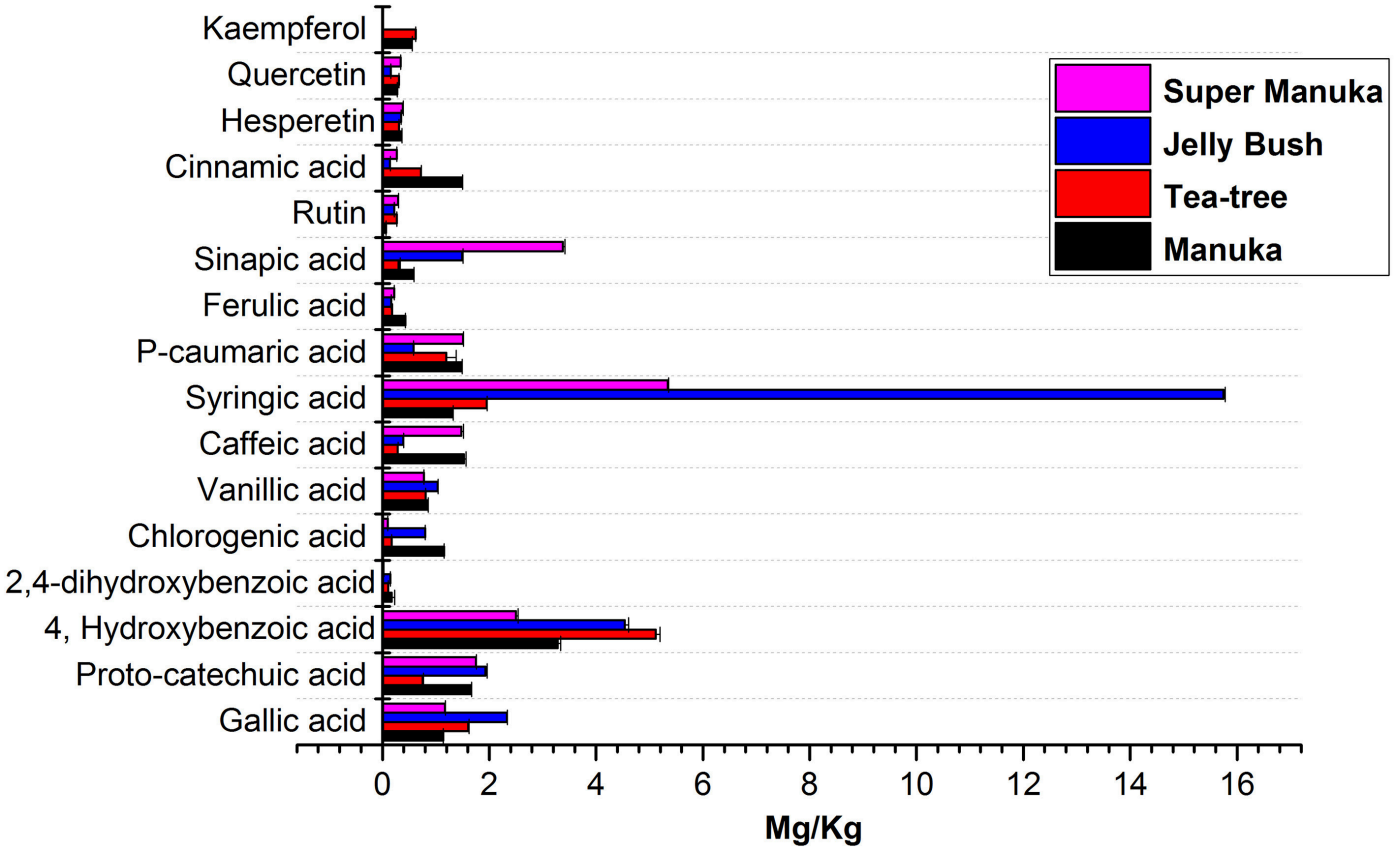
Comparative analysis of phenolic compounds in *Leptospernum* origin honeys.

**Figure 7:**
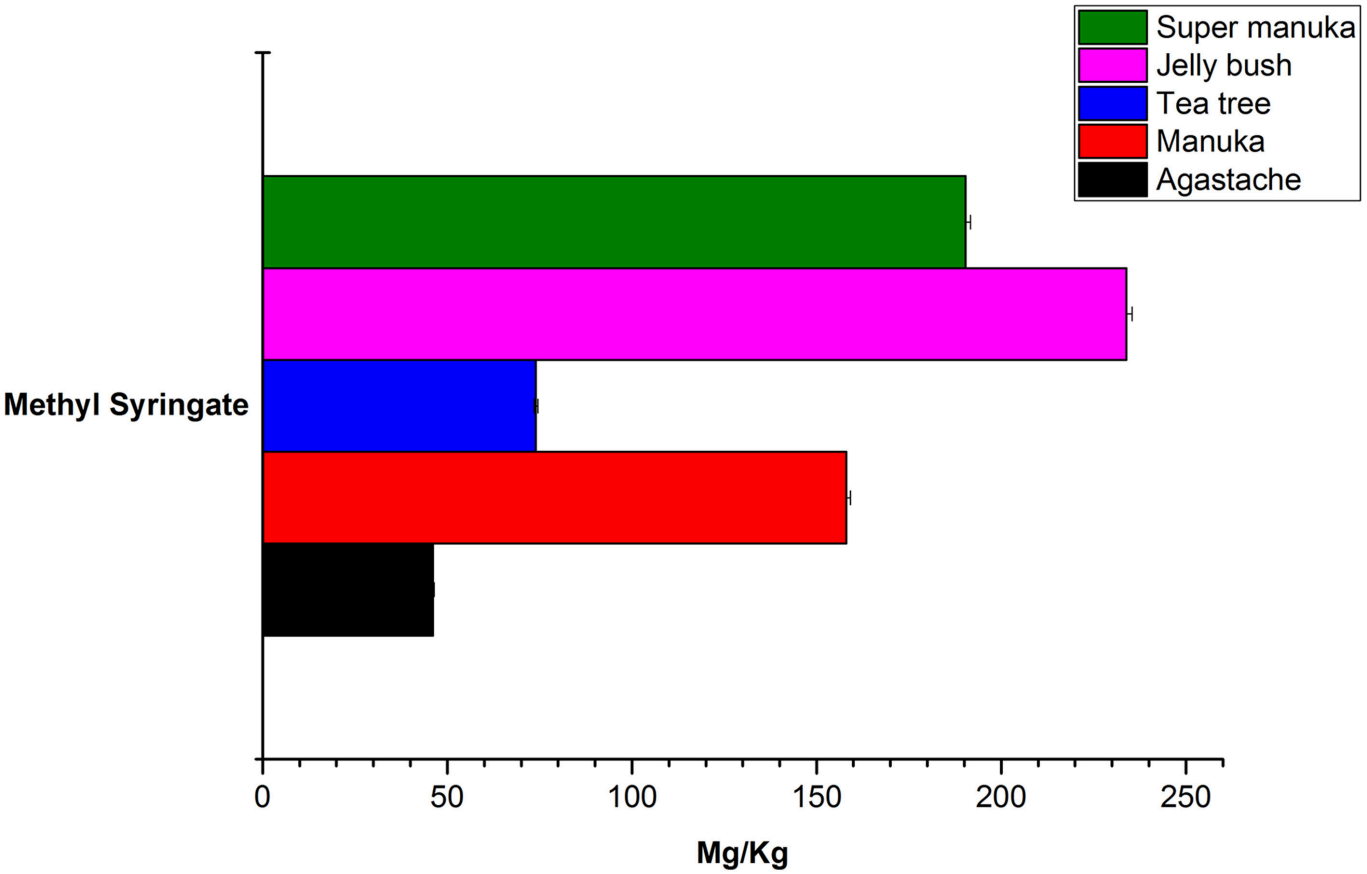
Comparative analysis of methyl syringate in honeys.

**Figure 8:**
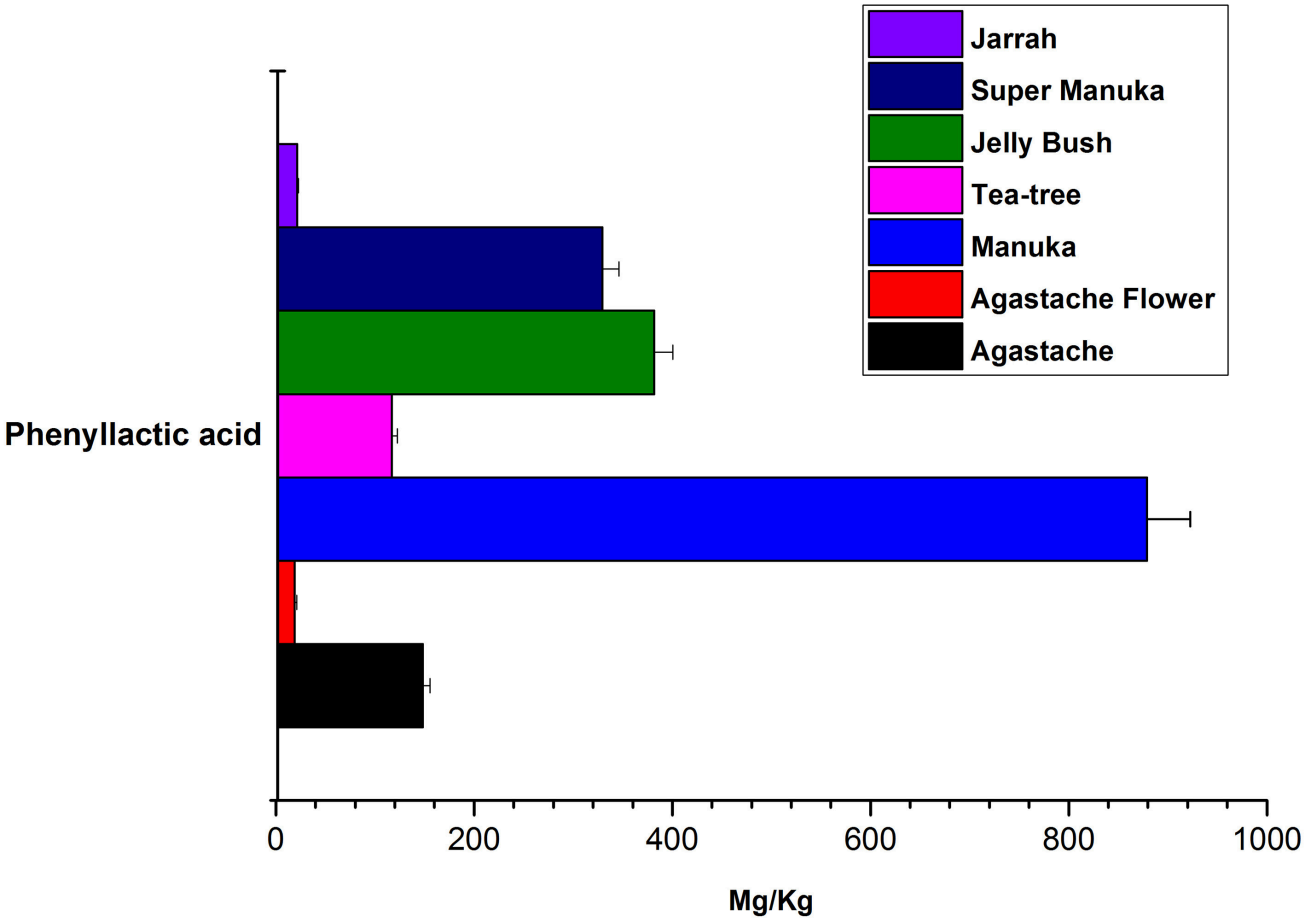
Comparative analysis of phenyllactic acid in honeys.

Jelly bush also belongs to the *Leptospermum* genus (tea trees) of which there are 80 types native to Australia. Manuka and Super manuka honeys are derived from trees of the *Leptospermum* genus found in New Zealand and Australia, respectively. Most compounds that were identified (except kaempferol) were present in variable amounts in all honeys of *Leptospermum* origin (Figures 6–8). Kaempferol was present only in Manuka and tea tree honeys. The results for methyl syringate and phenyllactic acid are presented in more detail (Figures 7, 8) as these two compounds were observed in higher amounts in these honeys. The levels of these two compounds in *Agastache* honey are included for comparison. *Agastache* honey also contains high concentrations of both of these compounds (Figures 7, 8). The quantification of phenolic compounds in these honeys is presented in Supplementary Table S1.

### Bioactive Compounds in Jarrah Honey

**Figure 9:**
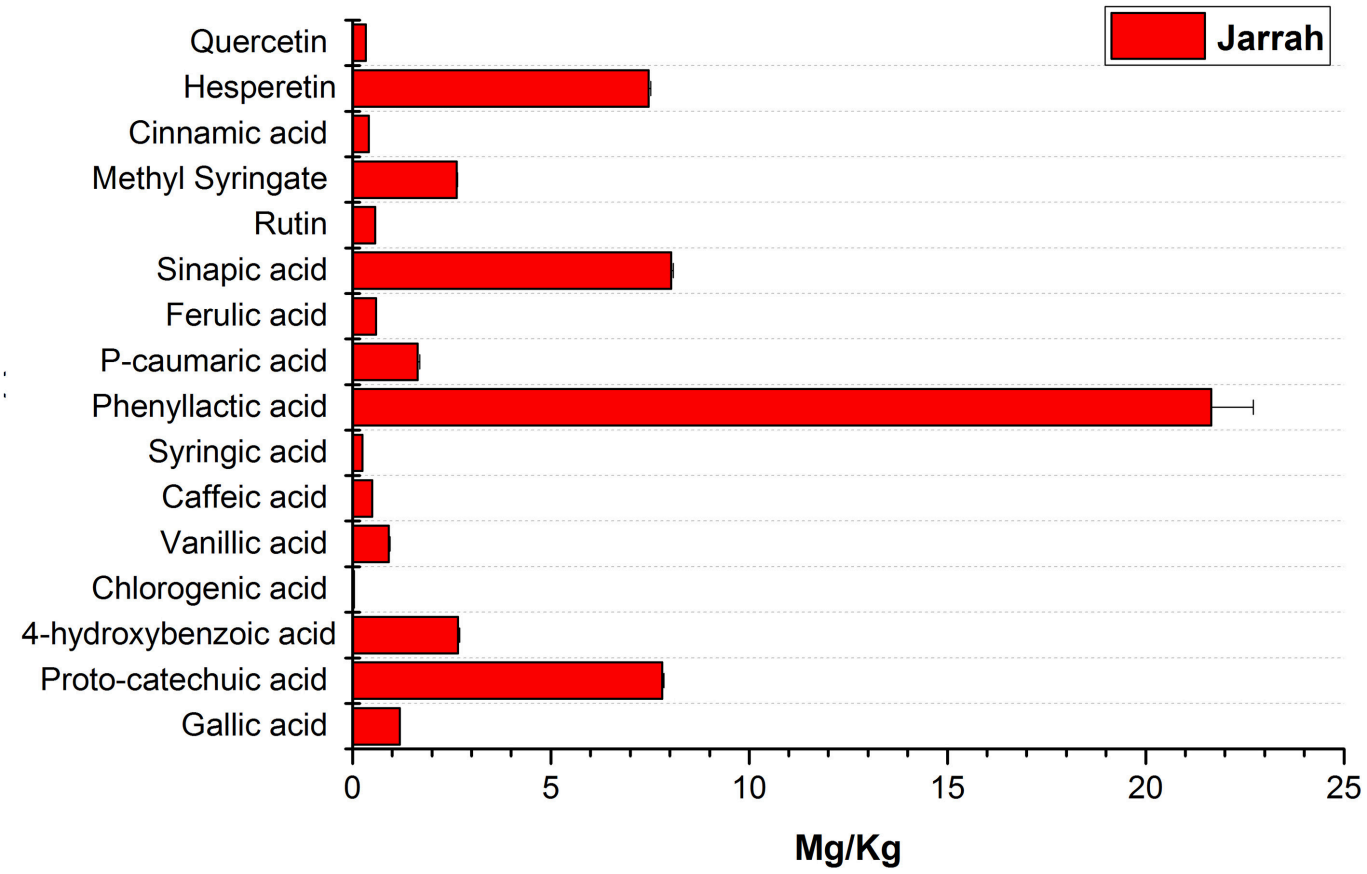
Phenolic profile of Jarrah honey.

The phenolic profile obtained for Jarrah honey (Figure 9) showed the presence of similar compounds to those found in *Agastache* and *Leptospermum* origin honeys, however, there were significant differences in the amount identified, for example, higher concentrations of hesperetin sinapic acid and proto-catechuic acid were observed in Jarrah honey than in *Agastache* and *Leptospermum* origin honeys, while the opposite was true for methyl syringate, phenyllactic acid, and 4-hydroxybenzoic acid.

## Discussion

Microbial resistance to currently available antimicrobial chemotherapeutic agents has become a major health problem worldwide (Ventola, 2015), necessitating the development of novel agents. Skin infections generally only require an antiseptic; however, some patients prefer to use a “natural” product in place of commercially available antiseptics for the management of local skin infections. We therefore evaluated honey as an alternative to antimicrobial and antiseptic application to infected wounds. In our study, the MIC and MBC values were consistent for the majority of honey/species combinations, indicating bactericidal activity of all honeys studied. All bacterial strains examined were susceptible to all honeys at concentrations ranging from 3.12 to 25% (w/v). There were generally no major differences between the susceptibilities of different members of the same species of bacteria. *Staphylococcus aureus* was the most susceptible of the three species examined and was the only species showing susceptibility at the level of 3.12% honey. *P. aeruginosa* was the least susceptible of the species examined, with susceptibility only achieved at 25% concentration of most honeys. *E. coli* was marginally more susceptible to most honeys than *P. aeruginosa*. In contrast to our findings, Tan et al. (2009), reported that *P. aeruginosa* was more susceptible than *E.coli* to Manuka honey, but at concentrations of 17.5 and 20%, respectively (Tan et al., 2009). Studies on Malaysian Tualang honey reported the efficacy of manuka honey was lower than that of Tualang honey against Gram negative bacteria in burn wound management (Nasir et al., 2010).

*Agastache* honey performed well against staphylococci (MIC/MBC 6.25/12.5), and was more effective than most other honeys (exception Tea tree honey) against *P. aeruginosa* (MIC/MBC 6.25/12.5). The efficacy of honeys of *Leptospermum* origin can be described in the descending order as follows: Tea tree > Jelly bush > Manuka > Super manuka. It is interesting that Super manuka honey required higher concentrations to inhibit staphylococci than Jelly bush honey although both are sourced from *Leptospermum polygalifolium*. Different manufacturing processes may account for this difference.

The bactericidal activity of *Agastache* honey was verified using confocal microscopy. There were a large number of non-viable cells in the treated samples compared to the untreated samples. The treated samples were analyzed after 24 h at variable concentrations of honey. At higher concentration (MIC/MBC), no intact cells were found, confirming the bactericidal activity of the honey. The effect of honey on the bacterial cells could be monitored by capturing images at different time intervals to estimate the time required to kill all cells.

The antimicrobial activity of the honey is due to the presence of various bioactive products, including hydrogen peroxide. Hydrogen peroxide did not contribute to the antimicrobial activity of Manuka honey or Jelly bush, but did contribute to the activity of the other honeys to variable degrees: Tea tree (35%), Super manuka (15%), Jarrah (12%), and *Agastache* honey (10%). Honeys derived from *Leptospermum* species (Manuka and Jelly bush) have activity based on methylglyoxal (MGO), the aldehyde form of pyruvic acid activity (Mavric et al., 2008); however, it is clear from the present study that MGO is not active in all honeys of *Leptospermum* origin.

### Common Bioactive Compounds in *Agastache* Honey and Flowers

The aim in identifying common phenolic compounds in *Agastache* honey and flowers was to determine whether significant compounds were transferred from flower nectar to the honey. The phenolic compounds reported were those which demonstrated pure peaks and a linear response. To declare the chemical marker, it is necessary to demonstrate high abundance and consistency in the honey. Nominating chemical markers provides an accurate and cost-effective method which can help differentiate one honey from another. In *Agastache* honey, the most abundant compounds observed were methyl syringate (46.09 ± 0.312 mg/kg) and phenyllactic acid (148.25 ± 7.27 mg/kg). Additional chemical markers observed in high abundance were 4-hydroxybenzoic acid (11.79 ± 0.197 mg/kg), *p*-caumaric acid and gallic acid. Therefore, predominance of these compounds may be a promising marker for authentication and determination of the origin of a particular honey.

In *Agastache* flower, the most abundant compounds were chlorogenic acid (198.09 ± 0.388 mg/kg), caffeic acid (39.23 ± 1.037 mg/kg), and rosmarinic acid. Some of the more common cinnamic acids were 4-caumaric acid, caffeic, ferulic, and sinapic acids. These can be found in a range of free forms and esterified forms e.g., with quinic acid as in chlorogenic acid. These compounds were apparently transferred in lower amounts from flower to the nectar, as they were not detected in the honey samples in high amounts.

Although methyl syringate was observed in high amounts in honey, flower contained only a low amount of this compound. To our knowledge, methyl syringate has never been reported to be present in plants of the *Lamiaceae* family, possibly because of low amounts present, although it is ubiquitous in other plant families. Flowers containing nectar could be the origin of this compound. Syringic acid was detected in both *Agastache* flower and honey. Although this compound is a common plant constituent, its methyl ester is rare. We suggest that the high amounts of methyl syringate found in *Agastache* honey could be explained by methyl esterification of syringic acid, although further investigation of this contention is required.

Rosmarinic acid was identified as a major compound in *Agastache* flower, consistent with the previous findings. Tuan et al. (2012) reported levels of 48.83 μg/g in *Agastache* flower, while Janicsák et al. (1999), comparing 96 *Lamiaceae* taxa, found that the concentration of rosmarinic acid was generally four times higher than caffeic acid. In the present study, rosmarinic acid was shown to be in high concentration in *Agastache* flower. Although the amount could not be quantified due to the non-linear response of the calibrators, a peak depicting a higher response for rosmarinic acid was observed (Supplementary Figure S1). The pure peak and higher response for both rosmarinic acid and caffeic acid suggests the value of these two compounds as chemical markers. Moreover, both compounds were also observed in *Agastache* honey, although at low concentrations. Other compounds (ferulic acid, syringic acid, vanillic acid, and proto-catechuic acid) that were also observed in higher amounts in flower than honey, confirm the production of these compounds by flowers and subsequently transferred through nectar to the honey.

The flavonoids kaempferol, quercetin, hesperetin, and rutin were present at low levels *in Agastache* honey and even lower levels in flowers. Previous studies indicated the presence of quercetin at low levels in *Agastache* flowers (Zielinska and Matkowski, 2014). During production of honey from nectar, some compounds might be modified in the bee-gut, but this process was not investigated in the present project.

### Leptospermum Honeys

The most abundant compound in *Leptospermum* honeys was phenyllactic acid. This compound was present in Manuka (878 mg/kg), Jelly bush (381.7 mg/kg), Super manuka (329.41 mg/kg), and Tea tree (117.12 mg/kg) honeys (Figure 6). These amounts could be overestimates as they were outside the range of calibrators; however, the compound was found in all *Leptospermum* origin honeys in higher amounts than in *Agastache* honey (148.25 mg/kg). Methyl syringate was also present in high amounts in *Leptospermum* honeys: Jelly bush (233.82 mg/kg), Super manuka (190.34 mg/kg), Manuka (158.08 mg/kg), and Tea tree (73.99 mg/kg). These levels are in the same range (155 to 288 mg/kg) as previously reported for Asphodel monofloral honey, sourced from *Asphodelus albus* (Tuberoso et al., 2009), but higher than that we found in *Agastache* honey (46.09 ± 0.312 mg/kg). Methyl syringate was also detected at low levels in robinia, rape, chestnut, clover, linden blossom, dandelion, sunflower, thyme and fir honeys (0.093 to 5.044 mg/kg) (Tan et al., 1990). Several authors reported the presence of methyl syringate in Manuka honey (Inoue et al., 2005), indeed Weston et al. found it to be the major phenolic component at 70% (w/w) (Weston et al., 2000).

Syringic acid was present in high concentrations in *Leptospermum* honeys: Jelly bush (15.74 mg/kg), Super manuka (5.34 mg/kg), Tea tree (1.95 mg/kg), and Manuka (1.31 mg/kg). Concentrations of 4-hydroxybenzoic acid were lower: Tea tree (5.11 mg/kg), Jelly bush (4.53 mg/kg), Manuka (3.28 mg/kg), and Super manuka (2.49 mg/kg). The levels of gallic acid found in our study were generally low: Jelly bush (2.32 mg/kg), Tea tree (1.60 mg/kg), Super manuka (1.16 mg/kg) and Manuka (1.13 mg/kg), in contrast to Yao et al. (2003) who reported higher levels (4.5 mg/100 g). Yao et al. (2003) also reported higher amounts of p-caumaric acid (0.05–4.74 mg/100 g) than found in the present study: Super manuka (1.51 mg/kg), Manuka (1.47 mg/kg), Tea tree (1.19 mg/kg), and Jelly bush (0.58 mg/kg). These differences are likely to be due to different production methods, climatic conditions or plant cultivars.

This suggestion is supported by the work of Oelschlaegel et al. (2012), who analyzed three groups of Manuka honeys characterized by different chemical markers: group 1, 4-hydroxybenzoic acid; group 2, Kojic acid and 2-methoxybenzoic acid; and group 3, syringic acid, 4-methoxyphenyllactic acid and methyl syringate. The results obtained in the present study corresponded to the group 3 results. Another study analyzed the flavonoid profile of 31 Manuka honeys and reported luteolin and chrysin were the principle components with quercetin and kaempferol at low levels (0.00 to 1.15 mg/kg). The present results for quercetin was in the range of 0.27–0.33 mg/kg which were consistent with previous reports (Chan et al., 2013). Hesperetin, keampferol, and rutin were present in low amounts. Therefore, the common chemical markers for *Leptospermum* origin honey are phenyllactic acid and methyl syringate. Importantly, the variable amount of syringic acid we found could help to differentiate Australian *Leptospermum origin* honeys from the New-Zealand Manuka honey.

### Jarrah Honey

The phenolic profile obtained for Jarrah honey identified the same chemical markers as found in *Agastache* and *Leptospermum* origin honeys; however, there were significant differences in the amounts identified. The amount quantified for the major compounds was as follows; phenyllactic acid (21.65 mg/kg), sinapic acid (8.02 mg/kg), proto-catechuic acid (7.81 Mg/kg), hesperetin (7.46 mg/kg), 4-hydroxybenzoic acid (2.65 mg/kg), and Methyl syringate (2.62 mg/kg). Martos et al. (2000b) reported that *Eucalyptus* honey contained myricetin, tricetin, quercetin, luteolin, and kaempferol. In the present study, quercetin was present in low amounts but, keampferol was not detected in the sample. Another study conducted on Turkish honey reported that *Eucalyptus* honey contained higher levels of proto-catechuic acid and low amounts of flavonoids (Kıvrak and Kıvrak, 2017) as observed in the present study. In the present study, hesperetin was the major flavonoid observed in Jarrah honey. It should be noted however that Jarrah is only one of many species of *Eucalyptus* native to Australia and the *Eucalyptus* honeys are likely to vary considerably in content, depending on the species as well as environmental factors. We suggest that the chemical markers for Jarrah honey are phenyllactic acid, sinapic acid, proto-catechuic acid and hesperetin.

### Bioactivity of Compounds

Many of the compounds identified in this study in *Agastache* and other honeys have been reported to have antimicrobial, antioxidant or anti-inflammatory activity suggesting the potential use of honey for the medical applications. Previous studies have shown the importance of some of these compounds in inhibiting bacterial growth and radical scavenging. All of the following compounds were found in varying concentrations in *Agastache honey* and in most of the other honeys that were examined in the present study.

Gallic acid is an anti-inflammatory compound and its derivatives are present in a number of phytomedicines with diverse biological and pharmacological activities, including radical scavenging, interfering with the cell signaling pathways and apoptosis of cancer cells (BenSaad et al., 2017). Proto-catechuic acid is considered to be an active component of some traditional Chinese herbal medicines such as *Cibotium barometz, Stenoloma chusanum, Ilex chinensis Sims*. It was reported to possess various pharmacological effects which may be closely correlated with its antioxidant activities. Similarly, 2,4-dihydroxybenzoic acid is an antioxidant compound (Kakkar and Bais, 2014). (+)-Catechin, a widespread plant bio-flavanoid, is a well-known antioxidant-free radical scavenger, reported as a component of green tea, as an anti-tumor agent and as an insect repellent (Bais et al., 2002).

4-hydroxybenzoic acid has been reported to have antimicrobial and fungicidal activity (Cho et al., 1998). Moreover, the esters of the conjugated compound with glycerol are antibacterial, inhibiting the growth of Gram-negative and Gram-positive bacteria (Kosová et al., 2015).

Chlorogenic acid, a natural chemical ester composed of caffeic acid and (-) quinic acid, has bactericidal activity against Gram negative bacteria (Kabir et al., 2014), while vanillic acid is active at low pH against many strains of *Listeria* (Delaquis et al., 2005). Caffeic acid and syringic acid possess both antimicrobial and antifungal activity at concentrations as low as 0.5 mg/ml (Chong et al., 2009). Another study conducted on the antioxidant and antimicrobial activities of caffeic acid, *p*-coumaric acid and rutin reported that these compounds inhibited the growth of *S. aureus* in chicken soup (Stojković et al., 2013). In addition to its antioxidant properties (Kikuzaki et al., 2002), sinapic acid, has antimicrobial and anti-inflammatory activity (Yun et al., 2008; Maddox et al., 2010; Nićiforović and Abramovič, 2014).

Cinnamic acid showed weak antibacterial activity against Gram-negative and Gram-positive bacteria, but was more effective against *Mycobacterium tuberculosis* (Guzman, 2014). Cinnamic acid also exhibited anti-fungal activity against *Aspergillus niger* and *Candida albicans* (Schmidt et al., 2010; Guzman, 2014).

Quercetin, is a well-known antioxidant (Rice-Evans et al., 1996), was also reported to be bacteriostatic against Gram positive bacteria (Wang et al., 2018). Hesperetin showed a wide range of antibacterial activity against *E. coli, S. aureus, S. epidermidis, Enterococcus faecalis, Salmonella typhimurium*, and *Enterobacter cloacae* when examined using the agar dilution method (Yi et al., 2008). Kaempferol, the major component of flowers of *Phytolacca octandra* exhibited dose dependent inhibition against clinical isolates of *E. coli* and *S. aureus* (Manivannan and Ilayaraja, 2013).

Phenyllactic acid is active against *S. aureus, E. faecalis*, and *Bacillus cereus*, and Gram-negative bacteria, such as *S. enterica, E. coli, Pseudomonas stuartii*, and *Klebsiella oxytoca* (Dieuleveux et al., 1998; Ohhira et al., 2004) as well as several yeasts (*C. pulcherrima, C. parapsilosis*, and *Rhodotorula mucilaginosa*) (Schwenninger et al., 2008; Mu et al., 2012).

The phenolic compounds reported to have antimicrobial activity and identified in *Agastache* honey were phenyllactic acid, 4-hydroxybenzoic acid, p-caumaric acid and gallic acid. In particular, methyl syringate should be investigated further as this compound was present in high amounts in *Agastache* honey. The compounds present in *Leptospermum* honeys with antimicrobial properties also included phenyllactic acid, methyl syringate, syringic acid, and 4-hydroxybenzoic acid, while Jarrah honey contained phenyllactic acid, 4-hydroxybenzoic acid as well as sinapic acid.

## Conclusions

In summary, our *Agastache* honey and commercially available medicinal honeys exhibited antimicrobial activity against skin-infection causing bacterial species, in particular *Staphylococcus aureus*. The loss of activity after treatment of some honeys with hydrogen peroxide suggests that hydrogen peroxide contributed to the antimicrobial activity of *Agastache*, Super manuka, and Jarrah honeys but not Manuka and Jelly bush honeys, while the activity of Tea tree honey was largely hydrogen peroxide based. A LC-MS/MS method developed for rapid quantification of 20 phenolic compounds provided an easy protocol to detect common medicinal compounds in the honey samples. *Agastache* honey contained several phenolic compounds which have been reported to have antimicrobial activity. The detection of the same compounds in Agastache honey and flowers indicated the source of bioactive compounds in the honey. The study also identified common compounds in honey samples of different origin, in particular phenyllactic acid and methyl syringate, as well as variable amounts of several compounds in different honeys, for example Australian *Leptospermum* origin honeys contained more syringic acid than manuka honey and *Agastache* honey (Figures 5, 6). *Leptospermum* honey products with a standardized level of antimicrobial activity have been developed in clinical practice for wound care. The present findings on the antimicrobial activity of *Agastache* honey are close to those of *Leptospermum* honeys, suggesting that *Agastache* honey products could be developed for the topical application against skin-infection causing bacteria and in wound care management. However, this needs to be further confirmed through *in vivo* and clinical studies.

## Author Contributions

NM, GL and EP conceptualized the project. SA, NM, and EP designed the experiments. SA performed microbiological testing. SA and PM performed LC-MS/MS experiments. SA, MD, NM, and EP analyzed the data. NM, EP, and GL contributed reagents, materials and analytical tools. SA wrote the draft manuscript. MD, NM, EP, and GL reviewed and approved the final manuscript.

### Conflict of Interest Statement

GL was employed by company Kenkay Pharmaceuticals. Kenkay Pharmaceuticals partially funded this work through Australian Research Council's Linkage Project scheme (Grant number LP120200743). The remaining authors declare that the research was conducted in the absence of any commercial or financial relationships that could be construed as a potential conflict of interest.
