# Supplementary material for: Antimicrobial Activity of Agastache Honey and Characterization of Its Bioactive Compounds in Comparison With Important Commercial Honeys
Source: Front Microbiol. 2019 Feb 25;10:263. doi: 10.3389/fmicb.2019.00263 (PMC6397887; doi:10.3389/fmicb.2019.00263)
Supplement: Supplementary file 1 [file Table_1.docx]

**Supplementary Table**

**Table S1.** The quantified amount of phenolic compounds in Six honeys and *Agastache* Flower.

| **Phenolic compounds  (Mg/kg)** | **Agastache** | **Agastache  flower** | **Manuka** | **Tea tree** | **Jelly bush** | **Super manuka** | **Jarrah** |
| --- | --- | --- | --- | --- | --- | --- | --- |
| **Gallic acid** | 1.75 ± 0.01 | 0.21±0.004 | 1.13±0.005 | 1.60±0.016 | 2.32±0.009 | 1.16±0.007 | 1.18±0.007 |
| **Proto-catechuic acid** | 1.90 ± 0.01 | 2.54 ± 0.013 | 1.66 ±0.007 | 0.75±0.013 | 1.92±0.034 | 1.75±0.01 | 7.81±0.04 |
| **4-hydroxybenzoic acid** | 11.79±0.197 | 3.84±0.064 | 3.28±0.055 | 5.11±0.085 | 4.53±0.076 | 2.49±0.042 | 2.65±0.04 |
| **Catechin** | 0.00 | 0.00 | 0.043 ± 0.02 | 0.00 | 0.065 ± 0.00 | 0.00 | 0.00 |
| **2,4- dihydroxybenzoic acid** | 0.35±0.010 | 0.27±0.001 | 0.17±0.058 | 0.10±0 | 0.14±0.007 | 0.00 | 0.00 |
| **Chlorogenic acid** | 1.18±0.002 | 198.09±0.388 | 1.15±0.002 | 0.17±0 | 0.79±0.002 | 0.10±0 | 0.04±0 |
| **Vanillic acid** | 1.65±0.008 | 3.21±0.016 | 0.85±0.004 | 0.80±0.004 | 1.03±0.005 | 0.77±0.004 | 0.91±0.036 |
| **Caffeic acid** | 0.59±0.015 | 39.23±1.037 | 1.52±0.040 | 0.28±0.007 | 0.38±0.010 | 1.47±0.038 | 0.49±0.013 |
| **Syringic acid** | 1.05±0.003 | 1.71±0.004 | 1.31±0.003 | 1.95±0.004 | 15.74±0.036 | 5.34±0.013 | 0.24±0.001 |
| **Phenyllactic acid** | 148.25±7.27 | 19.12±2.12 | 878.81±43.7 | 117.12±5.65 | 381.71±18.64 | 329.41±16.6 | 21.65±1.06 |
| **P-caumaric acid** | 3.97±0.015 | 2.43±0.009 | 1.47±0.015 | 1.19±0.185 | 0.58±0.002 | 1.51±0.005 | 1.64±0.047 |
| **Ferulic acid** | 0.39±0.002 | 1.19±0.010 | 0.43±0.001 | 0.18±0 | 0.16±0.001 | 0.22±0.001 | 0.59±0.002 |
| **Sinapic acid** | 1.18±0.008 | 0.33±0.002 | 0.58±0.004 | 0.29±0.031 | 1.49±0.014 | 3.37±0.043 | 8.02±0.058 |
| **Rutin** | 0.38±0.003 | 0.05±0 | 0.07±0.001 | 0.267±0.002 | 0.223±0.002 | 0.297±0.002 | 0.569±0.00 |
| **Methyl syringate** | 46.09±0.312 | 0.30±0.034 | 158.08±1.07 | 73.99±0.501 | 233.82±1.59 | 190.34±1.28 | 2.62±0.018 |
| **Cinnamic acid** | 0.88±0.013 | 0.20±0.001 | 1.49±0.002 | 0.71±0.012 | 0.14±0.006 | 0.26±0.001 | 0.40±0.003 |
| **Hesperetin** | 0.60±0.004 | 0.39±0.002 | 0.36±0.002 | 0.30±0.002 | 0.34±0.002 | 0.38±0.006 | 7.46±0.046 |
| **Quercetin** | 0.77±0.002 | 0.29±0.001 | 0.277±0.001 | 0.310±0.001 | 0.154±0.00 | 0.338±0.001 | 0.337±0.005 |
| **Kaempferol** | 1.55±0.004 | 0.57±0.002 | 0.55±0.002 | 0.62±0.002 | 0.00 | 0.00 | 0.00 |
