# Supplementary figures and images for: Antimicrobial Activity of Agastache Honey and Characterization of Its Bioactive Compounds in Comparison With Important Commercial Honeys
Source: Front Microbiol. 2019 Feb 25;10:263. doi: 10.3389/fmicb.2019.00263 (PMC6397887; doi:10.3389/fmicb.2019.00263)

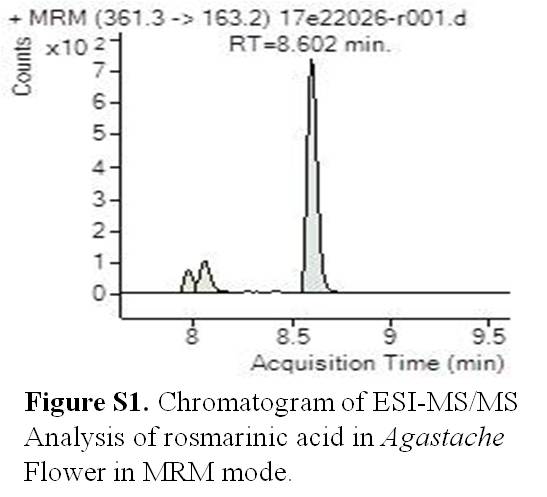

Supplement: Supplementary file 2 [file Image_1.jpg]
